# Aortoenteric Fistula Formation From Chronic Erosion of an Axios Gastroduodenal Stent in a Patient With a History of Radiation

**DOI:** 10.7759/cureus.80569

**Published:** 2025-03-14

**Authors:** Caleb M Glover, Adam Bowen, Claire Russell, Ali Rida, Alexandra Davies, Edward Cay, John Walling

**Affiliations:** 1 Internal Medicine, McLaren Greater Lansing, Lansing, USA; 2 Gastroenterology, McLaren Greater Lansing, Lansing, USA

**Keywords:** acute gi bleed, axios stenting, gastric outlet obstruction, primary aortoenteric fistula, self-expanding metal stents (sems)

## Abstract

Gastric outlet obstruction (GOO) secondary to malignant bowel obstruction (MBO) presents significant challenges in patients with advanced cancer, often impairing quality of life and nutritional intake. This report discusses a complex case of a 53-year-old male patient with stage IV colon cancer who developed an aortoenteric fistula (AEF) as a rare, life-threatening complication following duodenal stenting for GOO management. The case highlights the multifaceted etiology of AEF, involving prior radiation, chronic inflammation, and infected psoas abscess, culminating in massive gastrointestinal hemorrhage. While duodenal stents effectively restore luminal patency and alleviate symptoms in palliative settings, vigilant monitoring is crucial to promptly identifying and addressing potential complications, such as AEF. This underscores the need for a multidisciplinary approach in managing GOO in malignancy, balancing the benefits of self-expandable metallic stents (SEMS) against alternative interventions such as gastrojejunostomy (GJ), tailored to the patient's condition and prognosis to optimize outcomes and enhance quality of life.

## Introduction

Gastric outlet obstruction (GOO) secondary to malignant bowel obstruction (MBO) occurs when the passage of food from the stomach to the small intestine is blocked by tumor compression. Duodenal stents are commonly used to restore luminal patency and improve oral intake in palliative settings [1]. The most common complications of duodenal stents include stent migration, tumor ingrowth or overgrowth leading to obstruction, perforation, and gastrointestinal bleeding, with aortoenteric fistula (AEF) being an exceedingly rare yet devastating complication [2]. AEF is a life-threatening condition because it can cause massive gastrointestinal hemorrhage while also posing a high risk of sepsis due to the direct communication between the aorta and the gastrointestinal tract, necessitating urgent surgical intervention to prevent rapid deterioration and death. AEF as a complication of duodenal stenting is rare, with only a few documented cases [3-6].

## Case presentation

We present a 53-year-old male patient with a past medical history of type 1 diabetes mellitus, a history of pulmonary embolism (previously on apixaban), stage IV colon cancer status post right colectomy with lymph node resection, and duodenal stent placement one year earlier. The duodenal stent was placed secondary to significant scar formation from surgery and radiation therapy resulting in stenosis requiring stent placement. The patient's gastroduodenal stent was an Axios stent, a self-expanding, fully covered stent used to bypass strictures and blockages. It was placed via endoscopic ultrasound in the lesser curvature of the stomach to an arbitrary loop of the duodenum, which was adjacent to many small vessels. The patient was admitted for evaluation of worsening back pain, left lower extremity pain, and significant anemia. The anemia was identified during routine total parental nutrition (TPN)-related laboratory work (Table 1). Upon admission, he was noted to be drowsy, suspected to be secondary to narcotic burden for back pain management.

**Table 1:** Vital signs and laboratory investigations on admission

Parameter	Obtained value	Reference range
Temperature (°C)	37.8	36.1-37.2
Heart rate (beats/minute)	102	60-100
Blood pressure (mmHg)	100/65	90/60-120/80
Respiratory rate (breaths/minute)	18	12-20
Hemoglobin (g/dL)	6.5	13.5-17.5 (male)
White blood cell count (×10^9^/L)	15	4-11
Platelet count (×10^9^/L)	250	150-400
Serum creatinine (mg/dL)	1.2	0.6-1.2
Blood glucose (mg/dL)	180	70-130 (fasting)

**Figure 1:**
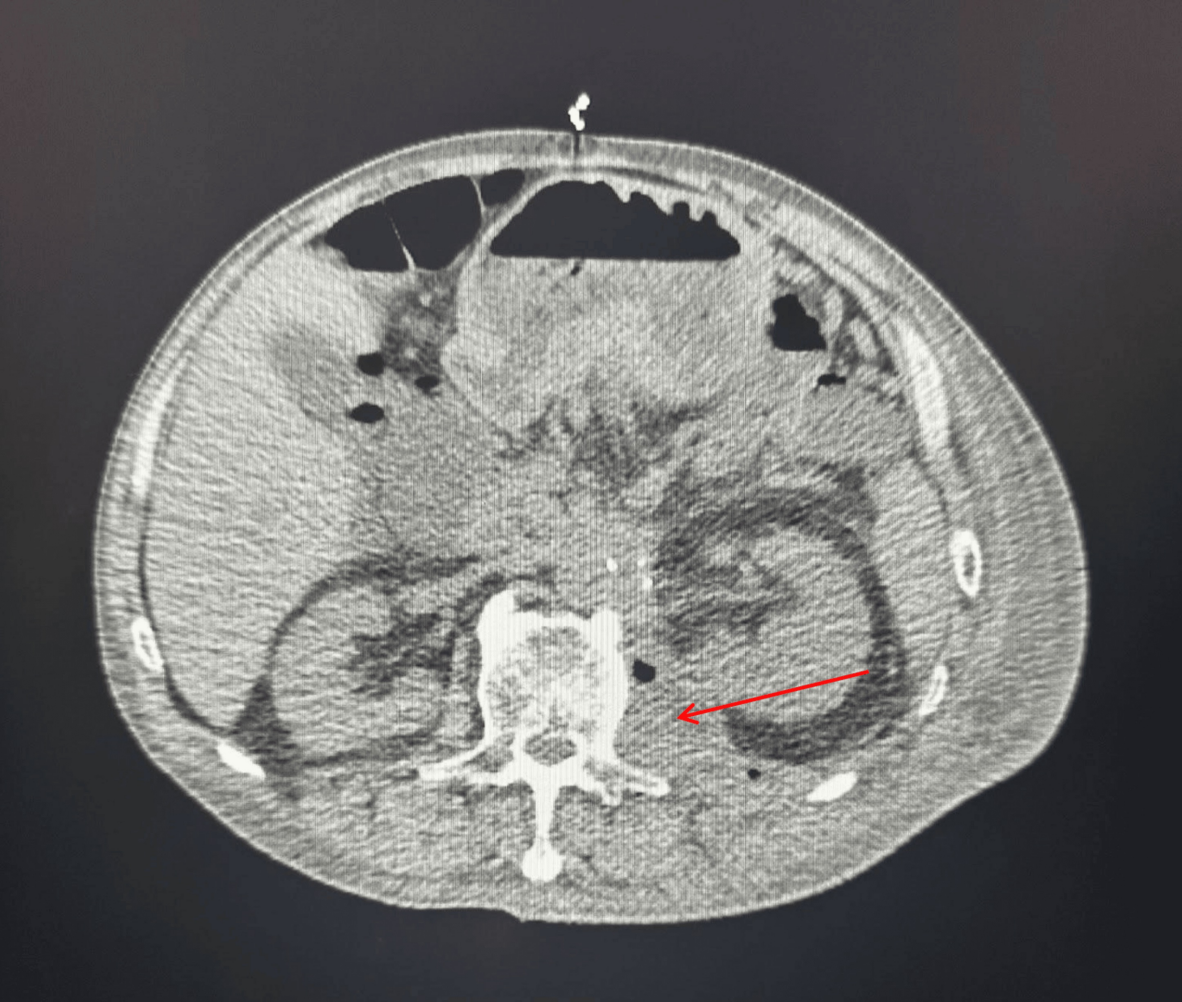
CT of the abdomen and pelvis demonstrating a poorly defined 6 × 3 cm fluid collection with air on the left psoas muscle consistent with psoas abscess (red arrow) CT: computed tomography

During his hospitalization, his medical course was complicated by a previously diagnosed small left psoas muscle abscess from a month prior to admission, for which he had been discharged on amoxicillin/clavulanic acid. Imaging during this admission revealed an enlarging air-containing fluid lesion within the left psoas, raising concern for a worsening abscess or infected hematoma (Figure 1). Cultures from his psoas abscess later grew multiple organisms, including group F streptococcus, *Escherichia coli*, *Klebsiella pneumoniae*, *Bacteroides ovatus*, and *Candida parapsilosis*. Given this, he was started on a prolonged course of piperacillin-tazobactam and anidulafungin while inpatient.

**Figure 2:**
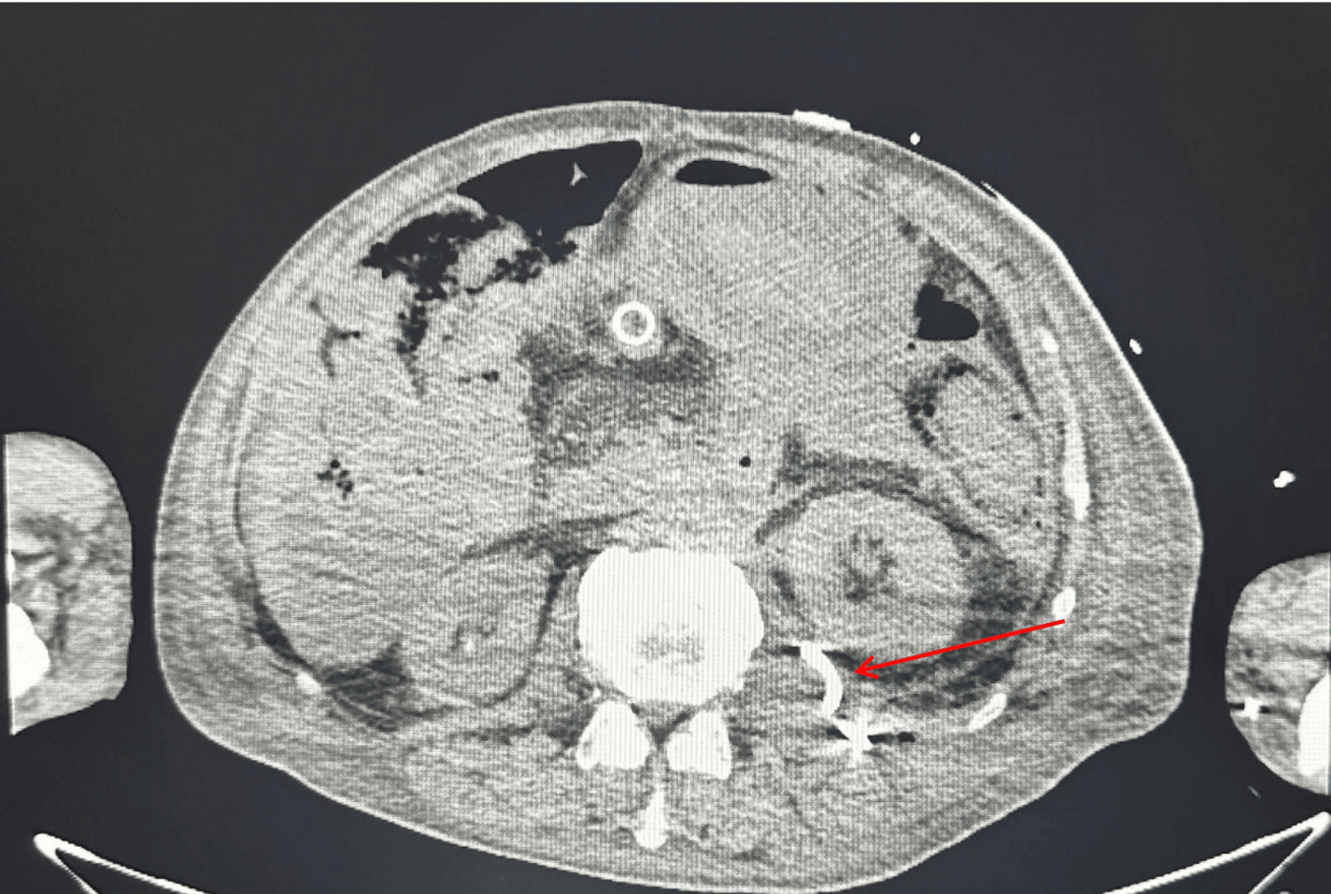
CT of the abdomen and pelvis demonstrating a poorly defined 6 × 3 cm fluid collection with air on the left psoas muscle consistent with psoas abscess following IR drain placement (red arrow) CT: computed tomography, IR: interventional radiology

**Figure 3:**
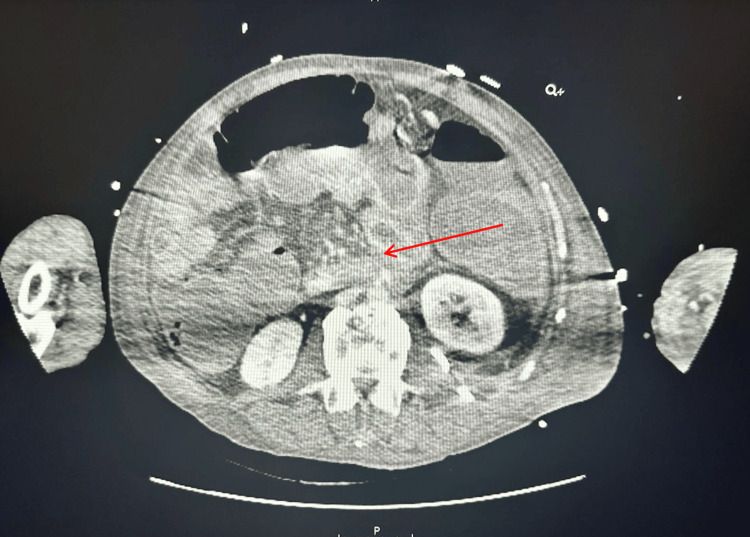
CT of the abdomen and pelvis demonstrating pooling of high-density contrast within the duodenum, which was suspicious for active hemorrhage within or near the duodenum (red arrow) CT: computed tomography

During his hospitalization, interventional radiology aspirated his psoas muscle abscess without signs of hemorrhage (Figure 2). Subsequently, he developed worsening, severe acute blood loss anemia secondary to hematemesis and hematochezia. He underwent esophagogastroduodenoscopy (EGD) evaluation with gastroenterology without evidence of active bleeding. The patient's status acutely worsened throughout the day, and a second endoscopic evaluation was performed with evidence of active bleeding in the duodenum. Computed tomography angiography of the abdomen was performed immediately before the EGD concerning for AEF as there was a confluence of vascular structures visualized near the duodenum (Figure 3). Vascular surgery was notified of the patient, and the patient was taken to the operating room emergently. The patient underwent exploratory laparotomy for the evaluation of the active bleed and possible AEF. Vascular surgeons placed an Omni Flush catheter in the distal aorta. Active extravasation and a saccular aneurysm 2-3 cm below the renal arteries were revealed. A prosthetic aortic cuff was successfully deployed, achieving hemostasis despite a small residual type 2 endoleak. The patient had a temporary abdominal closure system (ABThera) placed due to increased abdominal pressures. He remained intubated and sedated and was returned to the intensive care unit (ICU). On November 27, 2024, postoperative day 3 from endovascular aortic repair and decompressive laparotomy, the patient underwent a repeat exploratory laparotomy with fascial closure, during which his small bowel appeared viable, and no bowel was deemed necessary for resection. The patient was extubated shortly afterward and was successfully downgraded from the ICU to the medical floor, where he was discharged to subacute rehabilitation. The pathophysiology of the fistula was likely secondary to stent erosion into those vessels demonstrated on computed tomography.

## Discussion

GOO is a debilitating condition that arises when the distal stomach or proximal duodenum becomes obstructed. Patients with GOO typically present with progressive nausea, vomiting, early satiety, abdominal pain, and significant weight loss, leading to severe nutritional deficiencies and deterioration in overall health. While commonly associated with malignancies such as pancreatic and gastric cancers, GOO can also result from metastatic diseases, including advanced colorectal cancer [1]. In rare instances, colorectal cancer can metastasize to the stomach or surrounding structures, leading to GOO. GOO is relatively uncommon in colorectal cancer. According to a review on the management of intestinal obstruction in advanced malignancy, MBO occurs in approximately 10%-28.4% of colorectal cancer cases, but GOO is more uncommon [1]. The obstruction drastically impacts the quality of life by limiting oral intake, causing persistent discomfort, and often necessitating palliative interventions to restore gastric emptying and improve nutritional status. Effective management is essential to alleviate symptoms, enhance functional status, and support the patient's remaining lifespan.

Self-expandable metallic stents (SEMS) placed endoscopically or surgical/endoscopic gastrojejunostomy (GJ) are the primary treatment modalities. SEMS is commonly used to palliate malignant GOO in the setting of primary and metastatic cancer, offering rapid symptom relief and shorter hospital stays compared to GJ with similar rates of complications [7]. Complications of SEMS include bleeding, perforation, migration, occlusion, fistula formation, and in some instances, tumor ingrowth. Evidence-based recommendations suggest that SEMS is preferable for patients with limited life expectancy due to its minimally invasive nature and quicker recovery. At the same time, GJ may be more suitable for patients with a longer expected survival, as it is associated with a lower re-intervention rate. The complication rates of SEMS vary based on the study, with percent complication rate ranging between 0% and 45%, with stent dysfunction with obstruction or migration being the highest reported complications, followed by tumor ingrowth, with significant bleeding occurring in less than 1% of cases [2,6]. Stent types vary in design, with covered and uncovered options available. Covered stents are designed to prevent tumor ingrowth but may have a higher risk of migration, while uncovered stents have a lower migration risk but are susceptible to tumor ingrowth [8]. AEF is an exceedingly rare complication of stents for GOO, with only a few cases reported in the literature [2,4-6].

Primary AEFs occur de novo between the aorta and gastrointestinal tract without prior aortic interventions, often due to conditions such as aortic aneurysms or infections. In contrast, secondary AEFs develop as complications following surgical procedures, with aortic graft placements being the most represented cause [9]. The mortality rate associated with AEFs is notably high, often exceeding 50%, due to the potential for massive hemorrhage and sepsis. Management of AEFs typically involves prompt surgical intervention, with primary AEFs often requiring resection of the affected aortic segment and repair of the enteric defect. In contrast, secondary AEFs may necessitate graft removal and extensive debridement to address infection. Radiation therapy is a recognized risk factor for AEF formation, with studies documenting cases where patients developed AEFs following radiation treatment for thoracic malignancies [10]. Psoas abscesses can contribute to developing AEFs, particularly when the infection extends to adjacent structures, including the aorta or a duodenal stent in our patient's case, thereby increasing the risk of fistula formation [11].

## Conclusions

This case highlights the multifaceted and potentially life-threatening complications of gastric outlet obstruction (GOO) secondary to malignant bowel obstruction (MBO) in patients with advanced cancer, exemplified by the rare occurrence of aortoenteric fistula (AEF) following duodenal stenting. The patient's development of AEF, likely influenced by factors such as prior radiation, chronic inflammation, and an infected psoas abscess, emphasizes the critical need for vigilant monitoring after stent placement. The findings underscore the necessity for a tailored, multidisciplinary approach that balances the immediate benefits of self-expandable metallic stents (SEMS) against their risk of severe complications. Ultimately, this case reinforces the importance of personalized care strategies in enhancing patient outcomes and quality of life in individuals facing complex clinical scenarios related to advanced malignancies.
